# Alcohol Consumption and Lung Cancer Mortality in Japanese Men: Results from Japan Collaborative Cohort (JACC) Study

**DOI:** 10.2188/jea.16.49

**Published:** 2006-03-14

**Authors:** Yoshikazu Nishino, Kenji Wakai, Takaaki Kondo, Nao Seki, Yoshinori Ito, Koji Suzuki, Kotaro Ozasa, Yoshiyuki Watanabe, Masahiko Ando, Yoshitaka Tsubono, Ichiro Tsuji, Akiko Tamakoshi

**Affiliations:** 1 Division of Epidemiology, Miyagi Cancer Center Research Institute.; 2 Division of Epidemiology and Prevention, Aichi Cancer Center Research Institute.; 3 Department of Medical Technology, Nagoya University School of Health Sciences.; 4 Division of Public Health, Department of Infectious Disease Control and International Medicine, Niigata University Graduate School of Medical and Dental Science.; 5 Department of Public Health, Fujita Health University School of Health Sciences.; 6 Department of Epidemiology for Community Health and Medicine, Kyoto Prefectural University of Medicine Graduate School of Medical Science.; 7 Kyoto University Center for Student Health.; 8 Division of Health Policy, Tohoku University School of Public Policy.; 9 Division of Epidemiology, Department of Public Health and Forensic Medicine, Tohoku University Graduate School of Medicine.; 10 Department of Preventive Medicine/Biostatistics and Medical Decision Making, Nagoya University Graduate School of Medicine.

**Keywords:** Alcohol Drinking, Lung Neoplasms, Fruit, Vegetables, Cohort Studies

## Abstract

**BACKGROUND:**

The relationship between alcohol consumption and increased risk of lung cancer is controversial. This study was set up to investigate the association between alcohol consumption and death from lung cancer in a large Japanese cohort.

**METHODS:**

The subjects comprised 28,536 males, aged 40–79 years, living throughout Japan. During 268,464 person-years of follow-up, 377 lung cancer deaths were recorded. The hazard ratio (HR) of alcohol consumption for lung cancer mortality was calculated using the Cox proportional hazards model after adjustment for age, smoking and family history of lung cancer.

**RESULTS:**

There was no association between increased mortality from lung cancer and alcohol consumption among current drinkers. Compared with subjects who had never drunk alcohol, the HRs (95% confidence interval [CI]) of death from lung cancer for light (consuming <25.0 g ethanol per day), moderate (25.0–49.9 g per day) and heavy (≥50 g per day) drinkers were 0.81 (95% CI=0.61–1.07), 0.82 (0.61–1.11) and 0.97 (0.66–1.43), respectively. Further adjustment for fruit and vegetable intake did not change the results, and there was no change in HR materially after excluding those patients who died during the first 5 years of follow-up.

**CONCLUSIONS:**

These findings indicate that alcohol consumption was not associated with increased lung cancer mortality in this population of Japanese men.

Although alcohol is a risk factor for several sites of cancer,^1^ its relationship with lung cancer is still controversial. Recently, Bandera reviewed epidemiologic studies on alcohol consumption and lung cancer that presented smoking-adjusted risk estimates, and concluded that there might be an increased risk of lung cancer associated with drinking alcohol.^2^ On the other hand, a meta-analysis of the relationship between alcohol consumption and lung cancer risk by Korte presented a smoking-adjusted excess risk of lung cancer only in the very high alcohol consumption category (ethanol consumption was ≥2,000 g per month).^3^ These authors concluded that the results should be interpreted with caution due to the possibility that there was residual confounding in this group of subjects, and few studies have presented data on subjects who consumed 2,000+ g ethanol per month. Many of the previous studies on alcohol and lung cancer had problems with the methodology, including insufficient control for potential confounding factors such as smoking and dietary variables, and inappropriate grouping of subjects who had never drunk alcohol and ex-drinkers into a single referent category. Furthermore, most previous studies were conducted in Western countries, where the type of alcohol consumed and other drinking habits are quite different to those in Japan. In addition, individuals with the atypical allele of the aldehyde dehydrogenase 2 (ALDH2) gene, which results in inactive ALDH2 activity, bringing about a high blood concentration of acetaldehyde, the initial metabolite of alcohol, are prevalent in the Japanese population.^4^ Acetaldehyde has been shown to be carcinogenic in experimental animals,^5^ and therefore Japanese people may have a different susceptibility to lung cancer than Westerners.

This study was undertaken to examine the association between alcohol consumption and lung cancer mortality in a large Japanese cohort, with control for confounding factors and the separation of subjects who had never consumed alcohol from ex-drinkers.

## METHODS

### Study Cohort

The methodology of the baseline survey and follow-up in the JACC study, Japan Collaborative Cohort Study for Evaluation of Cancer Risk Sponsored by Monbusho (the Ministry of Education, Science, Sports and Culture of Japan) has been described in detail elsewhere.^6^ In brief, the established cohort members comprised 110,792 inhabitants (aged 40–79 years) of 45 study areas throughout Japan (46,465 men and 64,327 women). In most areas, individuals were selected from participants in municipal health checkups, and in other areas from whole populations or voluntary groups. Between 1988 and 1990, the individuals completed a self-administered questionnaire containing questions on medical history and lifestyle factors such as smoking, alcohol consumption, diet, physical activity and reproductive history. This study was approved by the Ethical Board of Nagoya University School of Medicine.

### Exposure Assessment

With regard to alcohol consumption, subjects were asked whether they were current or ex-drinkers, or had never drunk alcohol. Current drinkers were further asked about their drinking frequency, the type of beverage usually consumed, and the total amount drunk on a single occasion. The frequency of alcohol drinking was divided into four categories: less than once a week, once or twice a week, three or four times a week, and almost every day. For the beverage types usually consumed, subjects were asked to choose from five types (*sake*=rice wine, *shochu*=white spirits, beer, whisky and wine). However, the drinking frequency and amount consumed on a single occasion of each alcoholic beverage were not asked. The total amount consumed on a single occasion was converted by respondents into the corresponding equivalent of *sake* expressed as the traditional unit of *go* (1 *go* of *sake* = 180 mL, containing 22.8 g ethanol). In practice, the questionnaire presented the amount of alcoholic beverage which contained the same quantity of ethanol as 1 *go* of *sake*, and the subjects referred to this when recording the total amount consumed on a single occasion.

Average daily consumption of alcohol for each subject was calculated by multiplying the total amount consumed on a single occasion by the drinking frequency. Average daily consumption was then converted to the amount of ethanol in grams by multiplying the figure by 22.8.

The information on smoking habits was obtained by asking the subjects about their smoking status (current or former smoker, or never smoked), the age when they started smoking, and the average number of cigarettes smoked per day. Former smokers were also asked their age at cessation of smoking and/or length of time since they had stopped smoking. With regard to diet, the frequency of consumption of 33 common food items was asked (almost never, 1–2 times/month, 1–2 times/week, 3–4 times/week, and almost every day), in addition to the number of bowls of rice and miso-soup consumed per day.

### Follow-up

The vital and residential status of the subjects was followed up to December 31, 1999 by collation with residential registration in the various municipalities. Causes of death were ascertained from death certificates, which were coded according to the International Statistical Classification of Diseases and Related Health Problems, Tenth Revision (ICD-10).

Of the 46,465 original male cohort members, 1,557 subjects in two study areas were excluded because the questionnaire used in these districts did not include an item on drinking frequency. A further 4,412 subjects in four areas were also excluded because the questionnaire used there did not include an item on the amount of alcoholic beverage consumed on a single occasion, and a further 6,009 subjects in six areas were excluded because questions about past history of cancer and/or the frequency of consumption of green leafy vegetables, oranges, or fruit other than oranges were not included in the questionnaire used there. Finally, 27 subjects who reported a history of lung cancer in the questionnaire and 5,924 subjects with incomplete information about their drinking or smoking habits were also excluded. Consequently, the number of subjects evaluated in this study was 28,536. No analysis was conducted of female cohort members because their prevalence of daily drinking was low (4.7%).

The number of person-years of follow-up was calculated for each subject from the month of entry into the study until the month of death from any cause, the month of emigration outside the study area, or December 1999, whichever occurred first. A total of 268,464 person-years were accumulated and 377 lung cancer deaths (ICD-10=C34) were documented. A total of 883 subjects (3.1%) moving out of the study area during the follow-up period were identified from the residential registration; these individuals have been treated as censored cases.

### Statistical Analysis

Hazard ratios (HR) of lung cancer mortality in ex- and current drinkers compared with subjects who had never drunk alcohol was calculated, using the Cox proportional hazards regression model, employing the SAS^®^ PHREG procedure.^7^ HRs were adjusted for age, smoking and family history of lung cancer. Current drinkers were subdivided into three categories according to their daily ethanol consumption calculated from the baseline data, and the HR for each category was also assessed. In order to adjust for smoking status, current smokers were divided into six categories using the Brinkman Index (the number of cigarettes smoked per day multiplied by the number of years of smoking) as 1–399, 400–799, 800–1,199, 1,200–1,599, 1,600–1,999, and 2,000+, and ex-smokers into five categories according to how long they had stopped smoking: 0–4, 5–9, 10–14, 15–19 and 20+ years; these indices of smoking history were included in the model as dummy variables. The HR was also calculated with further adjustment for the frequency of consumption of green-leafy vegetables (1–2 times/week or less, 3–4 times/week, and almost every day), oranges and fruit other than oranges (1–2 times/month or less, 1–2 times/week, and 3–4 times/week or more), because a higher frequency of intake of green-leafy vegetables, oranges and fruit other than oranges was associated with a significant reduction in lung cancer mortality among males in this cohort^8^. Additionally, in order to examine the effect of including cases already diagnosed as lung cancer before the baseline survey, the HR was calculated after excluding cases that died in the first 5 years of follow-up. To assess effect modification by smoking, stratified analysis was conducted according to smoking status.

## RESULTS

Table 1 shows the baseline characteristics of subjects according to the level of alcohol consumption. The relative proportions of current drinkers, ex-drinkers and subjects who had never drunk alcohol were 73.1%, 6.8% and 20.0%, respectively. As alcohol consumption increased, the prevalence of individuals who had never smoked and those who ate vegetables and fruit every day tended to decrease. Among ex-drinkers, the prevalence of a history of stroke, myocardial infarction, liver disease and diabetes was higher than in the other groups.

**Table 1.** Baseline chacteristics of subjects by level of alcohol consumption.

	All Subjects	Never drinkers	Ever drinkers	Current drinkers (ethanol intake)			Ex-drinkers

				≤24.9 g/day	25.0-49.9 g/day	≥50.0 g/day	
Number of subjects	28,536	5,716	22,820	10,244	7,511	3,112	1,953

Mean age (year)	57.3	59.2	56.8	57.2	56.1	53.8	62.7

Smoking (%)							
Current	52.5	47.7	53.7	47.4	59.1	68.2	43.1
Past	26.4	22.6	27.3	28.5	26.0	19.1	39.6
Never	21.1	29.7	19.0	24.1	15.0	12.8	17.3

Daily dietary consumption (%)							
Green leafy vegetables	29.5	30.0	29.4	29.2	29.6	26.9	33.5
Oranges	26.6	33.4	24.9	27.2	23.1	18.1	31.3
Fruits other than oranges	27.2	32.5	26.0	28.1	24.1	19.6	31.9

Family history of lung cancer (%)	2.0	2.1	2.0	2.0	2.1	2.2	1.5

Past history (%)							
Stroke	2.0	2.0	2.0	1.7	1.3	0.8	7.9
Myocardial infarction	2.8	3.2	2.7	2.6	2.2	2.3	5.9
Liver disease	7.8	6.4	8.2	7.3	7.1	8.9	16.3
Diabetes	6.4	6.1	6.5	6.3	5.4	6.0	12.5

Table 2 presents the HR of lung cancer for drinkers compared with subjects who had never drunk alcohol. There was no association between current alcohol consumption and risk of lung cancer. After adjustment for age, smoking and family history of lung cancer, the HRs (95% confidence interval [CI]) were 0.81 (0.61–1.07) for those drinking <25.0 g ethanol per day, 0.82 (0.61–1.11) for those drinking 25.0–49.9 g per day, and 0.97 (0.66–1.43) for those drinking 50.0 g or more per day. The risk of lung cancer was also calculated for those drinking 66.7+ g per day (2,000+ g per month), and there was no clear risk elevation (HR=1.09, 95% CI=0.73–1.62). In contrast, the HR of lung cancer increased for ex-drinkers (HR=1.39, 95% CI=0.98–1.96). After further adjustment for the frequency of consumption of green-leafy vegetables, oranges and fruit other than oranges, the result did not change materially. The analysis was repeated after excluding cases that died in the first 5 years of follow-up; the HR for current drinkers was slightly elevated, but alcohol consumption was still not related to increased lung cancer mortality.

**Table 2.** Hazard ratio (HR)* of lung cancer by level of alcohol consumption.

	Person-years	No. of deaths	HR1	(95% CI)	HR2	(95% CI)	HR3	(95% CI)
Never drinkers	52,956	91	1.00	(reference)	1.00	(reference)	1.00	(reference)

Ever drinkers	215,508	286	0.90	(0.71-1.14)	0.96	(0.73-1.26)	1.03	(0.73-1.46)

Current drinkers (ethanol intake)								
≤24.9 g/day	97,334	113	0.81	(0.61-1.07)	0.81	(0.59-1.11)	0.90	(0.60-1.34)
25.0-49.9 g/day	71,863	85	0.82	(0.61-1.11)	0.90	(0.64-1.26)	0.99	(0.65-1.50)
≥50.0 g/day	29,679	38	0.97	(0.66-1.43)	0.98	(0.64-1.50)	1.08	(0.63-1.83)

Ex-drinkers	16,633	50	1.39	(0.98-1.96)	1.68	(1.16-2.45)	1.69	(1.03-2.76)

P for trend 1^†^			0.61		0.92		0.74	
P for trend 2^‡^			0.38		0.32		0.58	

* : HR1 is adjusted for age, smoking and family history of lung cancer. HR2 is further adjusted for intake of green-leafy vegetables, oranges and fruits other than oranges. HR3 means the relative risk with adjustment for the same covariates as those used for calculation of HR2 after cases who died in first five years of follow-up were excluded.

† : P for trend among current drinkers. The test for linear trends includes never drinkers.

‡ : P for trend among current drinkers. The test for linear trends excudes never drinkers.

CI: confidence interval

When stratified analysis according to smoking habits was performed, there was no significantly increased risk of lung cancer associated with current alcohol consumption, regardless of smoking status (Table 3). However, due to the small number of subjects in most categories, the statistical power of this analysis was limited.

**Table 3.** Hazard raito (HR)* of lung cancer by level of alcohol consumption according to smoking status.

		Never drinkers	Ever drinkers	Current drinkers (ethanol intake)			Ex-drinkers	P for trend 1^†^	P for trend 2^‡^

				≤24.9 g/day	25.0-49.9 g/day	≥50.0 g/day			
Never smokers	Person-years	13,368	36,575	21,297	9,607	3,331	2,339		
	No. of deaths	5	13	7	1	1	4		
	HR	1.00	1.22 (0.43-3.45)	1.10 (0.35-3.51)	0.37 (0.04-3.18)	1.15 (0.13-9.98)	4.20 (1.12-15.72)	0.61	0.54

Ex-smokers	Person-years	10,035	50,374	24,004	16,089	4,993	5,288		
	No. of deaths	19	61	27	15	2	17		
	HR	1.00	0.74 (0.44-1.25)	0.64 (0.36-1.16)	0.67 (0.34-1.33)	0.34 (0.08-1.47)	1.37 (0.71-2.64)	0.13	0.53
Current smokers (Cigarretes/day)									
≤20	Person-years	15,227	72,388	30,626	26,672	10,579	4,510		
	No. of deaths	33	110	44	35	16	15		
	HR	1.00	0.86 (0.58-1.27)	0.76 (0.48-1.20)	0.78 (0.48-1.27)	1.09 (0.59-2.01)	1.32 (0.72-2.43)	0.99	0.20

>20	Person-years	6,509	28,142	9,425	10,140	7,084	1,493		
	No. of deaths	12	57	11	23	13	10		
	HR	1.00	1.31 (0.69-2.47)	0.74 (0.33-1.70)	1.49 (0.73-3.03)	1.31 (0.58-2.93)	2.64 (1.13-6.14)	0.20	0.15

* : Adjusted for age, family history of lung cancer, intake of green-leafy vegetables, oranges and fruits other than oranges.

† : P for trend among current drinkers. The test for linear trends includes never drinkers.

‡ : P for trend among current drinkers. The test for linear trends excudes never drinkers.

95% confidence intervals in parentheses.

## DISCUSSION

No positive association between alcohol consumption and death from lung cancer was found in this large cohort of Japanese men, and the results were not modified by smoking status.

Alcohol is an established risk factor for several sites of cancer, and several potential biological mechanisms by which alcohol could increase cancer risk have been proposed, such as the carcinogenic effect of acetaldehyde and a reduction in the detoxification capacity of liver enzymes that metabolize carcinogens.^9^ However, results from previous prospective studies^10^^-^^18^ and case-control studies^19^^-^^27^ investigating the relationship between alcohol consumption and risk of lung cancer with adjustment for smoking status were not consistent. Several prospective^10^^-^^12^ and case-control^19^^-^^22^ studies showed an increased risk of lung cancer with alcohol consumption, and one case-control study presented only the risk stratified by smoking status and indicated an increased risk in heavy smokers.^23^ However, other studies failed to show any elevated risk of lung cancer with alcohol consumption.^13^^-^^18^^,^^24^^-^^27^ This inconsistency in results may be due to differences in the methodology used in the studies. Confounding by dietary variables and definition of reference category of alcohol consumption are important issues, in addition to residual confounding by smoking and misclassification of alcohol consumption. Certain dietary factors, especially fruit and vegetables, represent one possible factor that has consistently been shown to protect against lung cancer,^1^ and several investigations showed that the intake of fruit, vegetable, or related antioxidants was different according to drinking habits.^28^^-^^30^ However, only five studies have examined the risk adjusted for consumption of these foods or related antioxidants.^15^^,^^16^^,^^20^^,^^23^^,^^26^ In addition, ex-drinkers should be separated from those who have never drunk alcohol, and only the latter should be considered as the reference group, because ex-drinkers may quit drinking due to the effects of pre-clinical symptoms of lung cancer and thus show a higher incidence of lung cancer or mortality than those who have never drunk. Therefore, the use of nondrinkers, including both ex-drinkers and those who have never drunk alcohol, as the reference group may underestimate the effect of alcohol. However, only four of the previous studies separated ex-drinkers from subjects who had never drunk as the reference group.^14^^,^^20^^,^^22^^,^^25^

The present study had some merits compared with previous investigations that examined the relationship between alcohol and risk of lung cancer. This was a prospective study, so that various potential biases inherent in case-control studies were avoided. Adjustments were made for fruit and vegetable intake as potential confounding factors. Ex-drinkers were separated from subjects who had never drunk, and it was therefore possible to estimate the risk for drinkers compared with life-long abstainers. Adjustment for smoking history was performed not only for current smokers according to the amount and duration of smoking, but also for ex-smokers by recording the length of time they had stopped smoking.

The increase in lung cancer mortality in ex-drinkers found in this study may be due to inclusion of subjects who stopped drinking due to pre-clinical symptoms of lung cancer. However, after excluding cases who died in the first 5 years of follow-up, the overall results did not change. Ex-drinkers had different characteristics, such as a history of disease, from subjects who had never drunk, however, the HR for ex-drinkers did not change materially even after adjustment for a history of disease such as stroke, myocardial infarction, liver disease and diabetes (data not shown). Residual confounding by unknown factors could have contributed to the results. As no positive association between alcohol consumption and lung cancer mortality was observed in current drinkers, it is unlikely that a history of heavy drinking was the cause of an increase in lung cancer mortality in ex-drinkers.

The effect of alcohol consumption on lung cancer may differ according to the type of beverage consumed. Among previous studies investigating the effects of several different types of beverage, some reported an increased risk of lung cancer associated with the consumption of beer,^20^^,^^23^ hard liquor,^11^^,^^26^ or both.^12^ The present study did not investigate the association between any particular type of alcoholic beverage and lung cancer mortality because the questionnaire asked subjects about all types of alcoholic beverage consumed by selection from five types, but did not allow quantification of the amount of each beverage consumed. In this cohort, 71.5% of current drinkers consumed *sake* (rice wine), and 52.1% consumed beer. To our knowledge, the effect of *sake* on lung cancer has not been investigated in previous studies, so that it is not clear whether the fact that approximately two-thirds of current drinkers in this cohort usually consumed *sake* affected the results of this study. There were relatively few drinkers who consumed beverages with a high ethanol concentration, such as whisky and *shochu*. The proportion of whisky drinkers and *shochu* drinkers was 17.8% and 19.0%, respectively. A recent prospective study suggested that wine has a protective effect against lung cancer,^12^ although the proportion of wine drinkers in the present cohort was low (2.7%), and so the effect of wine consumption in this study would have been small.

Death from lung cancer was used as the end point, and this may have affected the results of this study for the following reasons: first, some of the subjects may have suffered from lung cancer at the baseline survey and changed their alcohol consumption due to the disease. However, the HR for current drinkers compared with those who had never drunk did not change materially after excluding cases who died in the first 5 years of follow-up. Second, lung cancer cases were only censored at the time of death, so that the impact of alcohol on the prognosis may have been reflected on the result. However, since the survival rate of patients with lung cancer was low,^31^ the association between alcohol consumption and risk of lung cancer may not be distorted substantially.

Alcohol consumption at baseline was used as the marker of alcohol exposure, and past alcohol consumption was not considered. Therefore, evaluation of exposure at a single time point may have diluted the true effect of alcohol on lung cancer. However, duration of alcohol consumption, another important indicator of alcohol exposure, was not associated with lung cancer mortality (data not shown).

In conclusion, this JACC study found no association between alcohol intake and death from lung cancer. The results do not support the hypothesis that there is a relationship between alcohol intake and increased risk of lung cancer.

## References

[1] World Cancer Research Fund and American Institute for Cancer Research. Food, nutrition and the prevention of cancer: a global perspective. American Institute for Cancer Research. Washington, D.C., 1997. 10.1016/s0899-9007(99)00021-0 10378216

[2] Bandera EV, Freudenheim JL, Vena JE Alcohol consumption and lung cancer: a review of the epidemiologic evidence. Cancer Epidemiol Biomarkers Prev 2001; 10: 813-21. 11489747

[3] Korte JE, Brennan P, Henley SJ, Boffetta P Dose-specific meta-analysis and sensitivity analysis of the relation between alcohol consumption and lung cancer risk. Am J Epidemiol 2002; 155: 496-506. 11882523 10.1093/aje/155.6.496

[4] Takeshita T, Morimoto K, Mao XQ, Hashimoto T, Furuyama J Phenotypic differences in low K_m_ aldehyde dehydrogenase in Japanese workers. Lancet 1993; 341: 837-8. 8096045

[5] Woutersen RA, Appelman LM, Van Garderen-Hoetmer A, Feron VJ Inhalation toxicity of acetaldehyde in rats. III. Carcinogenicity study. Toxicology 1986; 41: 213-31. 3764943 10.1016/0300-483x(86)90201-5

[6] Ohno Y, Tamakoshi A Japan collaborative cohort study for evaluation of cancer risk sponsored by Monbusho (JACC Study). J Epidemiol 2001; 11: 144-50. 11512570 10.2188/jea.11.144 PMC11735075

[7] SAS/STAT software: changes and enhancements, release 6.11. SAS Institute, Cary, NC, 1996.

[8] Ozasa K, Watanabe Y, Ito Y, Suzuki K, Tamakoshi A, Seki N, Dietary habits and risk of lung cancer death in a large-scale cohort study (JACC Study) in Japan by sex and smoking habit. Jpn J Cancer Res 2001; 92: 1259-69. 11749690 10.1111/j.1349-7006.2001.tb02148.x PMC5926679

[9] Blot WJ Invited commentary: more evidence of increased risks of cancer among alcohol drinkers. Am J Epidemiol 1999; 150: 1138-40. 10588074 10.1093/oxfordjournals.aje.a009939

[10] Klatsky AL, Friedman GD, Siegelaub AB Alcohol and mortality. A ten-year Kaiser-Permanente experience. Ann Intern Med 1981; 95: 139-45. 7258861 10.7326/0003-4819-95-2-139

[11] Pollack ES, Nomura AM, Heilbrun LK, Stemmermann GN, Green SB Prospective study of alcohol consumption and cancer. N Engl J Med 1984; 310: 617-21. 6694673 10.1056/NEJM198403083101003

[12] Prescott E, Gronbaek M, Becker U, Sorensen TI Alcohol intake and the risk of lung cancer: influence of type of alcoholic beverage. Am J Epidemiol 1999; 149: 463-70. 10067906 10.1093/oxfordjournals.aje.a009834

[13] Kvale G, Bjelke E, Gart JJ Dietary habits and lung cancer risk. Int J cancer 1983; 31: 397-405. 6832851 10.1002/ijc.2910310402

[14] Kono S, Ikeda M, Tokudome S, Nishizumi M, Kuratsune M Alcohol and mortality: a cohort study of male Japanese physicians. Int J Epidemiol 1986; 15: 527-32. 3818161 10.1093/ije/15.4.527

[15] Bandera EV, Freudenheim JL, Marshall JR, Zielezny M, Priore RL, Brasure J, Diet and alcohol consumption and lung cancer risk in the New York State Cohort (United States). Cancer Causes Control 1997; 8: 828-40. 9427425 10.1023/a:1018456127018

[16] Woodson K, Albanes D, Tangrea JA, Rautalahti M, Virtamo J, Taylor PR Association between alcohol and lung cancer in the alpha-tocopherol, beta-carotene cancer prevention study in Finland. Cancer Causes Control 1999; 10: 219-26. 10454067 10.1023/a:1008911624785

[17] Breslow RA, Graubard BI, Sinha R, Subar AF Diet and lung cancer mortality: a 1987 National Health Interview Survey cohort study. Cancer Causes Control 2000; 11: 419-31. 10877335 10.1023/a:1008996208313

[18] Djousse L, Dorgan JF, Zhang Y, Schatzkin A, Hood M, D’Agostino RB, Alcohol consumption and risk of lung cancer: the Framingham Study. J Natl Cancer Inst 2002; 94: 1877-82. 12488481 10.1093/jnci/94.24.1877

[19] Koo LC Dietary habits and lung cancer risk among Chinese females in Hong Kong who never smoked. Nutr Cancer 1988; 11: 155-72. 2841651 10.1080/01635588809513983

[20] De Stefani E, Correa P, Fierro L, Fontham ET, Chen V, Zavala D The effect of alcohol on the risk of lung cancer in Uruguay. Cancer Epidemol Biomarkers Prev 1993; 2: 21-6. 8380549

[21] Murata M, Takayama K, Choi BC, Pak AW A nested case-control study on alcohol drinking, tobacco smoking and cancer. Cancer Detect Prev 1996; 20: 557-65. 8939341

[22] Dosemeci M, Gokmen I, Unsal M, Hayes RB, Blair A Tobacco, alcohol use, and risks of laryngeal and lung cancer by subsite and histologic type in Turkey. Cancer Causes Control 1997; 8: 729-37. 9328195 10.1023/a:1018479304728

[23] Bandera EV, Freudenheim JL, Graham S, Marshall JR, Haughey BP, Swanson M, Alcohol consumption and lung cancer in white males. Cancer Causes Control 1992; 3: 361-9. 1617124 10.1007/BF00146890

[24] Kabat GC, Wynder EL Lung cancer in nonsmokers. Cancer 1984; 53: 1214-21. 6692309 10.1002/1097-0142(19840301)53:5<1214::aid-cncr2820530532>3.0.co;2-8

[25] Restrepo HE, Correa P, Haenszel W, Brinton LA, Franco A A case-control study of tobacco-related cancers in Colombia. Bull Pan Am Health Organ 1989; 23: 405-13. 2611462

[26] Carpenter CL, Morgenstern H, London SJ Alcoholic beverage consumption and lung cancer risk among residents of Los Angels County. J Nutr 1998; 128: 694-700. 9521630 10.1093/jn/128.4.694

[27] Zang EA, Wynder EL Reevaluation of the confounding effect of cigarette smoking on the relationship between alcohol use and lung cancer risk, with larynx cancer used as a positive control. Prev Med 2001; 32: 359-70. 11304097 10.1006/pmed.2000.0818

[28] Kesse E, Clavel-Chapelon F, Slimani N, van Liere M; E3N Group Do eating habits differ according to alcohol consumption? Results of a study of the French cohort of the European Prospective Investigation into Cancer and Nutrition (E3N-EPIC). Am J Clin Nutr 2001; 74: 322-7. 11522555 10.1093/ajcn/74.3.322

[29] Barefoot JC, Gronbaek M, Feaganes JR, McPherson RS, Williams RB, Siegler IC Alcoholic beverage preference, diet, and health habits in the UNC Alumni Heart Study. Am J Clin Nutr 2002; 76: 466-72. 12145024 10.1093/ajcn/76.2.466

[30] Ruidavets JB, Bataille V, Dallongeville J, Simon C, Bingham A, Amouyel P, Alcohol intake and diet in France, the prominent role of lifestyle. Eur Heart J 2004; 25: 1153-62. 15231374 10.1016/j.ehj.2003.12.022

[31] Ajiki W, Matsuda T, Sato Y, Fujita M, Yamazaki S, Murakami R, A standard method of calculating survival rates in population-based cancer registries. Jpn J Cancer Clin 1998; 44: 981-93. (In Japanese)

